# Management and safety of somatostatin receptor ligands and pegvisomant in pregnant women with acromegaly: a narrative review of the literature

**DOI:** 10.1007/s12020-026-04761-x

**Published:** 2026-08-21

**Authors:** Margherita Medici, Valentino Marino Picciola, Irene Comune, Alessandro Cicale, Maria Chiara Zatelli, Maria Rosaria Ambrosio

**Affiliations:** 1 Section of Endocrinology, Geriatrics and Internal Medicine, Department of Medical Sciences, University of Ferrara, Via Ariosto 35, Ferrara, 44124 Ferrara Italy; 2 Endocrine Unit, University Hospital S. Anna, Ferrara, 44124 Italy

**Keywords:** Acromegaly, Pituitary Adenoma, Pregnancy, Somatostatin receptor ligands, Pegvisomant, Fetal outcomes

## Abstract

**Purpose:**

Pregnancy in acromegaly is a rare event. Its management is still a challenge given the lack of data, the risk of tumor progression and maternal-fetal complications. This study aims to evaluate the impact of different treatment strategies, particularly somatostatin receptor ligands (SRL) and pegvisomant (PEG), on maternal and neonatal outcomes through a comprehensive review of the literature.

**Methods:**

We performed a narrative review of the literature. A PubMed search was performed using the keywords “acromegaly AND pregnancy”, combined with treatment-related terms, covering publications from January 1, 1988, to January 21, 2026. No publication type restrictions were applied. Additional articles were identified through manual reference screening. Articles published in English and Italian were included.

**Results:**

A total of 44 articles were identified, comprising 120 patients and 134 pregnancies; we further contribute an additional case of pregnancy with pasireotide exposure, representing the third reported case of pasireotide exposure during pregnancy. Medical management was categorized as follows: treatment withdrawal before conception (*n* = 40), discontinuation upon pregnancy confirmation (*n* = 47), and continuation or modification throughout gestation (*n* = 30). Symptom worsening occurred in 16.7–40%. Maternal complications included gestational diabetes (5.6–18.8%) and pre-eclampsia (2.8–10.3%). Overall, fetal outcomes were favorable, with only one congenital malformation. Breastfeeding was successfully achieved in most cases.

**Conclusion:**

Pregnancy in acromegaly is generally characterized by clinical stability, supporting the safety of treatment withdrawal upon conception in most patients. While early therapy discontinuation is standard, SRL and/or PEG treatment is a viable option for patients requiring tumor and biochemical control. Individualized multidisciplinary management is essential.

## Introduction

Acromegaly is a rare endocrine disorder caused by growth hormone (GH) hypersecretion, usually due to a pituitary adenoma (PA) with an estimated incidence of approximately 10 cases per million individuals [1]. Pregnancy in women with acromegaly has historically been uncommon, with fewer than 200 cases reported in the literature up to 2019 [2]. This rarity is mainly explained by the high prevalence of menstrual irregularity, affecting up to 81% of women with acromegaly, and by the relatively advanced age at diagnosis (45 ± 3.04 years at diagnosis), both of which significantly limit reproductive potential [3, 4]. Infertility is also frequently observed among women of reproductive age with acromegaly [5]. Despite these reproductive challenges, the occurrence of pregnancy in women with acromegaly appears to be increasing, likely because of improved disease control and the wider use of assisted reproductive techniques; nevertheless, acromegaly during pregnancy remains a rare clinical condition [6]. Historically, women with acromegaly were often advised against pregnancy due to concerns regarding potential loss of biochemical disease control, tumor progression, and the possible adverse effects of elevated maternal GH levels on fetal development [2]. Physiological pituitary enlargement during pregnancy, particularly in the presence of an intrasellar PA, may increase the risk of compressive symptoms, especially in women with macroadenomas [6]. However, in most cases, pregnancy is associated with hormonal and tumor stability [6]. When compressive symptoms occur, they do not necessarily indicate true tumor progression, but may instead reflect physiological pituitary enlargement, tumor re-expansion following treatment withdrawal, or, less frequently, pituitary apoplexy [6]. From a maternal perspective, pregnancy in acromegaly was associated with worsening of hypertensive status. Overall, pregnancy-related blood pressure deterioration was observed in 45% of cases, rarely progressing to preeclampsia or eclampsia [7]. Conversely, the rates of worsening diabetes, preterm delivery, miscarriage, small-for-gestational-age infants, and congenital anomalies are all lower than those observed in the general pregnant population [8]. Management of acromegaly during pregnancy remains challenging. Transsphenoidal surgery (TSS) may be considered prior to conception in women planning pregnancy, whereas during gestation it is reserved for cases with significant tumor compression or neurological symptoms [6]. Owing to limited safety data, long-acting somatostatin analogues are generally discontinued, with a possible switch to short-acting octreotide before conception, and medical therapy is typically stopped once pregnancy is confirmed, being resumed only in selected cases with severe symptoms or tumor-related complications. Due to scarce evidence, pasireotide is not recommended during pregnancy [9]. Data from prolactinoma patients indicate that cabergoline is safe for fetal development, mitigating concerns regarding its use in acromegaly [10]. In this context, the present review aims to analyze the effects of somatostatin receptor ligands (SRL) and pegvisomant (PEG) on maternal and fetal outcomes in pregnant women with acromegaly.

## Methods

We conducted a narrative review of the literature. A comprehensive literature search was conducted in PubMed, covering publications from 1st January 1988 to 21st January 2026. The primary search term was “acromegaly AND pregnancy”, which was combined with specific treatment-related keywords to enhance sensitivity, including pasireotide, octreotide, lanreotide, cabergoline, and PEG Although cabergoline is not a somatostatin analog, it was included in the search strategy to broaden the scope and increase the likelihood of identifying eligible studies. No publication type restrictions were applied. We included studies published in English or Italian involving pregnant women diagnosed with acromegaly, regardless of gestational age or maternal age. The diagnosis could have been established either before conception or during pregnancy. Eligible studies reported on pharmacological treatment with SRL (octreotide, lanreotide, pasireotide) or PEG, administered at any dose, by any route, and for any duration throughout pregnancy. Studies were considered for inclusion if they reported at least one of the following primary outcomes: maternal disease control during pregnancy, evaluated through clinical symptoms and serum IGF-1 levels; and fetal or neonatal safety outcomes, including congenital malformations, spontaneous abortion, preterm birth, or other neonatal complications. Studies were excluded only if they did not report original clinical data or failed to provide extractable details on maternal disease control or pregnancy/neonatal outcomes. Additionally, we checked the reference lists of the included studies and performed a backwards citation analysis. Three reviewers independently deduplicated the results, screened titles and abstracts against the inclusion criteria, and retrieved the full texts of potentially eligible studies. Full-text assessments were also conducted independently by the reviewers. Any disagreements were resolved through discussion with a fourth author. Data from the included studies were systematically collected using a Microsoft Excel spreadsheet specifically designed to standardize and facilitate the extraction of relevant information. For each selected article, the following variables were recorded: bibliographic details (first author, year of publication), study characteristics (case report, case series, cohort, etc.), number of patients, details related to the diagnosis of acromegaly, type of pharmacological treatment administered during pregnancy (including drug, dosage, duration, and route of administration), timing of diagnosis (preconception or during pregnancy), maternal outcomes (clinical and biochemical disease control), and fetal or neonatal outcomes (spontaneous abortion, preterm delivery, congenital malformations, and neonatal complications). All data were manually extracted and cross-checked to ensure accuracy and consistency with the original publications.

## Results

### Population characteristics and study selection

The review identified 44 articles, comprising 120 patients and 134 pregnancies [2, 5, 7, 11–51].

We further contribute an additional case of pregnancy with pasireotide exposure, representing the third reported case of pasireotide exposure during pregnancy [7, 50]. This case involves a 35-year-old woman with acromegaly who had been treated with pasireotide during the early phase of pregnancy. The diagnosis of acromegaly, attributed to a pituitary macroadenoma, was established six years before the onset of pregnancy. Initial medical management consisted of a first-generation SRL, specifically long-acting octreotide (20 mg/28 days), which demonstrated suboptimal biochemical control, despite dose escalation to 30 mg/28 days (insulin-like growth factor 1, IGF-1 780 ng/mL, 2.66 ULN). Subsequently, transsphenoidal surgical resection of the macroadenoma was performed. Histopathological analysis revealed low proliferative activity (Ki-67 < 3%). After surgery, due to persistent disease activity and lack of response to first-generation SRL, pasireotide therapy was initiated, with progressive dose titration from 40 mg/28 days to 60 mg/28 days. Cabergoline (1.5 mg/week) was subsequently added to the regimen and adequate biochemical disease control was reached. Serial magnetic resonance imaging (MRI) demonstrated a small, stable intrasellar adenomatous residue, far from the optic chiasm. Upon confirmation of an unexpected pregnancy at 5 weeks of gestation, all medical therapies were discontinued. Throughout the gestational period, the patient remained asymptomatic with respect to acromegaly and arterial hypertension; however, in the late third trimester, she reported a moderate headache, coincident with an episode of influenza. IGF-1 levels were 218 ng/mL (0.78 ULN), 298 ng/ml (1.07 ULN) and 391 ng/mL (1.41 ULN) in the first, second, and third trimesters, respectively, consistent with expected trimester-specific values considering the progressive contribution of placental GH. Serial visual field assessments remained within normal limits. During the early gestational period, impaired fasting glucose (IFG) was identified, likely attributable to recent pasireotide administration. Thereafter, gestational diabetes mellitus (GDM) was diagnosed based on oral glucose tolerance testing. Glycemic control and glycated albumin levels (< 15%) were maintained within target ranges with nutritional therapy alone until 35 weeks of gestation, at which point insulin therapy was initiated. In the first trimester, a retrochorionic hematoma was observed via transvaginal ultrasonography. Fetal growth remained consistently appropriate for gestational age, with a trend towards the upper limits of normal during the third trimester. At 38 weeks’ gestation, labor was medically induced following premature rupture of membranes and meconium-stained amniotic fluid. Due to abnormalities detected during cardiotocographic monitoring, an emergency cesarean section was performed. At birth, the neonate’s weight (3520 g) and length (50.5 cm) were within normal ranges (P83 and P75, respectively, according to Italian Neonatal Studies [INeS] references), with no evidence of congenital malformations. The APGAR scores were 9 at 1 min and 10 at 5 min post-partum. However, at 10 min, respiratory distress was observed, necessitating admission to the neonatal intensive care unit. Pneumothorax and pneumomediastinum were identified, considered likely unrelated to maternal pathology, and resolved within five days. Three weeks after delivery, the patient manifested headache, periorbital edema, hyperhidrosis. Biochemical evaluation revealed elevated IGF-1 (1085 ng/mL, 3.9 ULN) and GH (3.99 ng/mL) levels. MRI imaging demonstrated a millimetric increase in the intrasellar adenomatous residue. Medical therapy with pasireotide and cabergoline was subsequently reinitiated, resulting in clinical improvement and reduction in IGF-1 levels (185.5 ng/mL, 0.67 ULN). Breastfeeding was discontinued.

Including this additional case, the final pooled cohort consisted of 121 patients and 135 pregnancies (14 patients with two pregnancies [5, 7, 24, 26, 30, 35, 38, 39, 46, 49]).

The literature predominantly relies on case reports (*n* = 27) [11, 12, 15–22, 25–29, 31–34, 36, 37, 42, 44, 45, 47, 50, 51], retrospective studies (*n* = 10) [2, 5, 7, 13, 23, 24, 30, 40, 43, 48] and case series (*n* = 6) [14, 35, 39, 41, 46, 49]. Only one prospective study was identified [38]. The main clinical characteristics and pregnancy outcomes of the included patients are summarized in Table 1.

**Table 1:** Main characteristics and pregnancy outcomes of patients in the included studies

Study (Author, Year)	Pregnancies (*n*)	Mean age at diagnoses	Mean age at pregnancy	Pre-gestational diagnosis (*n*)	Macro / Micro (*n*)	Medications at conception (*n*)	Continued/Resumed therapy during pregnancy (*n*)	Obstetric Complications (*n*)		Pregnancy & Fetal Outcomes
								GDM	PE	
Our Case	1	29	35	1	1/0	1 (PAS + CAB = 1)	0	1	0	None
Assal A. et al., 2016	4	23,33	33,67	4	2/0	2 (OCT = 2)	1 (OCT = 1)	0	0	None
Atmaca A. et al., 2006	1	23	24	1	1/0	1 (OCT = 1)	0	-	-	1 TToP
Babinska A. et al., 2021	1	32	-	1	1/0	1 (SRL I gen = 1)	1 (SRL I gen = 1)	0	0	None
Brian SR. et al., 2007	1	26	-	1	1/0	1 (PEG = 1)	1 (PEG = 1)	0	0	None
Caron P. et al., 2010	14	-	-	14	11/2	-	-	1	1	4 SGA
Cheng S. et al., 2012	11	29	-	11	5/2	3 (OCT = 1; SRL I gen + DA = 1; SRL I gen + PEG = 1;	3 (OCT = 1; SRL I gen + DA = 1; SRL I gen + PEG = 1;	0	0	1 Twin Pregnancy
Cheng V. et al., 2012	1	30	30	0	-	0	1 (OCT = 1)	-	-	None
Colao A. et al., 1997	4	25	31	4	-	3 (OCT = 3)	1 (OCT = 1)	-	-	None
Cozzi R. et al., 2006	5	-	32,8	5	4/0	2 (OCT = 2)	0	0	0	None
Das L. et al., 2021	1	30	30	0	1/0	0	1 (OCT = 1)	0	0	None
De Menis E. et al., 1999	1	29	-	1	1/0	1 (LAN = 1)	0	0	0	None
Dias M. et al., 2014	10	-	31,2	10	9/0	8 (OCT = 4; PEG = 1; SRL I gen + DA = 3)	0	1	1	1 LBW
Dicuonzo F. et al., 2019	1	29	36	1	1/0	1 (OCT = 1)	0	0	0	None
Dogansen SC. et al., 2018	2	26,5	31,5	2	2/0	2 (SRL I gen = 1; SRL I gen + DA = 1)	2 (BRC = 2)	1	0	2 SAB
Fassnacht M. et al., 2001	1	24	-	1	1/0	1 (SRL I gen + DA = 1)	1 (OCT = 1)	-	-	None
Guarda FJ. et al., 2020	4	25,5	35,25	4	3/0	2 (SRL I gen + PEG = 1; DA + PEG = 1)	0	2	0	1 Twin Pregnancy; 1 LBW
Haliloglu O. et al., 2016	5	-	-	5	5/0	5 (SRL I gen + DA = 3; OCT = 1; LAN = 1)	0	-	-	None
Hannon AM. et al., 2019 (a)	1	32	32	0	1/0	0	1 (OCT = 1)	1	0	None
Hannon AM. et al., 2019 (b)	4	-	-	4	3/1	4 (OCT = 3: SRL I gen + DA = 1)	1 (CAB = 1)	0	0	None
Herman-Bonert V. et al.,1998	3	29,67	32	3	3/0	2 (OCT = 2)	0	-	-	None
Hierl T. et al., 2000	1	36	-	1	1/0	1 (OCT = 1)	0	-	-	None
Jallad RS. et al., 2018	15	28,08	32,53	15	12/0	11 (OCT = 8; SRL I gen + PEG = 1; PASI = 1; SRL I gen + DA = 1)	11 (OCT = 10; PAS = 1)	1	5	1 SAB; 2 LBW; 1 Ureteral stenosis.
Jiao R. et al., 2023	6	22,2	28	6	4/0	0	0	1	0	1 TToP; 1 SAB
Kasuki L. et al., 2012	1	26	-	1	1/0	1 (OCT = 1)	0	0	0	None
Kim SK. et al., 2010	1	27	34	1	-	1 (OCT = 1)	0	0	0	None
Landolt A., 1989	1	-	37	1	1/0	1 (OCT = 1)	0	0	0	None
Lau SL. et al., 2008	1	19	-	1	1/0	1 (LAN = 1)	0	-	-	None
Maffei P. et al., 2010	1	25	36	1	0/1	1 (OCT = 1)	1 (OCT = 1)	0	0	None
Mikhail N., 2002	1	29	30	1	-	1 (OCT = 1)	1 (OCT = 1)	-	-	None
Montini M. et al., 1990	1	36	37	1	1/0	1 (SRL I gen + DA = 1)	0	1	0	None
Mozas J. et al., 1999	1	27	27	1	1/0	1 (OCT = 1)	1 ( OCT = 1)	0	0	None
Neal JM., 2000	1	40	43	1	1/0	1 (OCT = 1)	1 (OCT = 1)	-	0	None
Önder E. et al., 2017	5	29,2	34	5	5/0	5 (SRL I gen + DA = 3; OCT = 2)	0	0	0	1 TToP
Petyt M. et al., 2023	1	21	-	1	1/0	1 (PAS = 1)	0	0	0	None
Pirchio R. et al., 2023	12	26,89	32,58	12	8/1	2 (SRL I gen = 2)	2 (SRL I gen = 2)	1	0	1 SAB
Qureshi A. et al., 2006	1	29	-	1	1/0	1 (PEG = 1)	0	1	0	None
Serri O. et al., 2003	1	33	34	1	0/1	1 (OCT = 1)	0	0	0	None
Shimatsu A. et al., 2010	1	36	-	1	1/0	1 (OCT = 1)	0	0	0	None
Takano T. et al., 2006	2	24	31,5	2	-	2 (OCT = 2)	0	0	0	None
Takeuchi K. et al., 1999	1	36	36	0	1/0	0	1 (OCT = 1)	-	0	None
Teltayev D. et al., 2017	1	22	24	1	1/0	1 (LAN = 1)	0	0	0	None
Torun AN. et al., 2012	1	28	-	1	1/0	1 (OCT = 1)	1 (OCT = 1)	0	0	None
Wiesli P. et al., 2006	1	31	37	1	1/0	0	0	0	0	None
Tan JY. et al., 2025	1	26	31	1	1/0	1 (DA + PEG = 1)	1 (PEG = 1)	0	0	None

**Macro**, Pituitary adenoma *≥* 1 cm; **Micro**, Pituitary adenoma < 1 cm; **BRC**, bromocriptine; **CAB**, cabergoline; **DA**, dopamine agonists; **GDM**, gestational diabetes mellitus; **LBW**, low birth weight (< 2500 g); **OCT**, octreotide; **PAS**, pasireotide; **PE**, preeclampsia; **PEG**, pegvisomant; **SAB**, spontaneous abortion; **SGA**, small for gestational age; **SRL**, somatostatin receptor ligands; **TToP**, therapeutic termination of pregnancy. Blank cells indicate that the corresponding data were not available

### Baseline patient characteristics

Acromegaly was diagnosed prior to conception in most cases (97.04%; 131/135 pregnancies) [Our Case, 2, 5, 7, 11–16, 18–35, 37–44, 46, 47, 49–51] and during pregnancy in the remaining 2.96% (4/135) [17, 36, 45, 48]. Macroadenomas were prevalent (92.59%; 100/108) [Our Case, 5, 7, 11, 12, 14–20, 23–25, 27–30, 33, 34, 37, 38, 40–45, 45–48, 48, 50, 51]. Regarding reproductive history, menstrual irregularities were common, with amenorrhea reported in 38.64% of evaluable patients (*n* = 17/44) [12, 14, 18, 19, 22, 23, 25, 29, 32, 33, 39, 42, 46]. The mean maternal age at conception was 32.31 ± 4.73 years (range 24–43). Most pregnancies (89.13%; 82/92) occurred spontaneously [Our Case, 7, 11, 12, 14–19, 21–24, 26, 28, 29, 31–39, 41, 42, 44, 47, 48, 50], while 8.7% (8/92) followed assisted reproductive technology (ART) [20, 25, 27, 46, 49, 51] and 2.17% (2/92) followed ovulation induction [38, 49]. Two twin pregnancies were documented [35, 46]. Baseline characteristics are summarized in Table 2.

**Table 2:** Baseline characteristics of the study population

Category	Feature	Value
Study Cohort	Total Patients	121
	Total Pregnancies	135
	Patients with 2 pregnancies	14
Acro-Diagnosis	Mean age at diagnosis (years)	27.77 ± 5.60 (15–41)
	Diagnosed before conception	97.04% (131/135) *
	Diagnosed during pregnancy	2.96% (4/135) *
Tumor Features	Macroadenoma (≥ 1 cm)	92.59% (100/108) *
	Microadenoma (< 1 cm)	7.41% (8/108) *
Previous Therapy	Transsphenoidal surgery	85.96% (98/114) *
	Radiotherapy	20.72% (23/111) *
Reproductive Data	Mean age at conception (years)	32.31 ± 4.73 (24–43)
	**Conception Mode** (*n* = 92) *	
	- Spontaneous	89.13% (*n* = 82)
	- ART (IVF/ICSI/IUI)	8.70% (*n* = 8)
	- Ovulation induction	2.17% (*n* = 2)

*Percentages were calculated based on the total number of cases for which specific data were available in the included literature. **IVF**, In Vitro Fertilization; **ICSI**, Intracytoplasmic Sperm Injection; **IUI**, Intrauterine Insemination

### Management of acromegaly diagnosed during pregnancy

Acromegaly was diagnosed *de novo* during pregnancy in 2.96% of cases (*n* = 4), with a mean age at diagnosis of 32.0 ± 2.83 years [17, 36, 45, 48]. Diagnosis typically occurred in the first trimester (range 11–13 weeks) [17, 36, 45], although one case was identified in the second trimester [48]. Clinical presentation was dominated by severe headache [36, 45] and visual field defects [17, 48], associated with typical acromegalic features [17, 45]. Diagnosis was confirmed biochemically (elevated IGF-1 with a reported range of 373–755 ng/mL) [17, 36, 48] and neuroradiologically (macroadenomas in 3 available cases) [17, 45, 48]. No patient underwent neurosurgery or radiotherapy during pregnancy. Management involved first-generation SRL - either short-acting subcutaneous octreotide [17, 36, 45] or octreotide LAR [48] - initiated between the 13th and 20th weeks of gestation and continued until delivery. SRL therapy generally controlled symptoms, though one patient required dose escalation due to tumor expansion and developed GDM [45]. Pregnancy outcomes were favorable in all cases. All neonates (*n* = 4) were healthy, with a mean birth weight of 3160.75 ± 248.73 g and none reported malformations [17, 36, 45, 48]. In two cases, breastfeeding was avoided to continue medical therapy [17, 45]. Conversely, one patient treated with octreotide LAR breastfed for 4 months without adverse neonatal effects [48].

### Management of acromegaly diagnosed prior to pregnancy

The largest cohort consisted of 117 patients (131 pregnancies) diagnosed with acromegaly prior to conception [Our Case, 2, 5, 7, 11–16, 18–35, 37–44, 46, 47, 49–51]. Detailed data regarding pre-conception surgical and radiotherapy treatments are summarized in Table 2. Medical therapy strategies were categorized into three groups, based on management decisions during pregnancy: group 1, pre-conception withdrawal of acromegaly therapy; group 2, discontinuation of acromegaly therapy upon pregnancy diagnosis; group 3, continuation, modification, or re-introduction of acromegaly therapy during pregnancy. Notably, 14 pregnancies from one multicenter study were excluded from categorical analysis due to aggregated data [30]. Detailed obstetric and neonatal outcomes for each group are summarized in Table 3.

**Table 3:** Maternal and neonatal outcomes according to medical therapy management strategy

Outcome	Feature	Group 1 (*n* = 40)	Group 2 (*n* = 47)	Group 3 (*n* = 30) **
Clinical Course	Symptom Worsening	26.67% (4/15) *	16.67% (6/36) *	40% (8/20) *
	Symptom Improvement	6.67% (1/15) *	11.11% (4/36) *	0% (0/20) *
Maternal Complications	Gestational Diabetes Mellitus	5.56% (2/36) *	18.75% (6/32) *	12.00% (3/25) *
	Pre-eclampsia	5.40% (2/37) *	2.78% (1/36) *	10.34% (3/29) *
Pregnancy Outcome	Live Births	90.63% (29/32) *	95.65% (44/46) *	88.89% (24/27) *
	Spontaneous Abortion	6.25% (2/32) *	0% (0/46) *	11.11% (3/27) *
	Therapeutic Abortion	3.13% (1/32) *	4.35% (2/46) *	0% (0/27) *
	Perinatal Mortality	0%	0%	0%
Delivery Data	Term Delivery	94.44% (17/18) *	88.89% (32/36) *	95.00% (19/20) *
	Pre-term Delivery	5.56% (1/18) *	8.33% (3/36) *	5.00% (1/20) *
	Post-term Delivery	0% (0/18) *	2.78% (1/36) *	0% (0/20) *
Delivery Mode	Spontaneous Vaginal	47.06% (8/17) *	41.67% (10/24) *	31.58% (6/19) *
	Elective Cesarean	52.94% (9/17) *	45.83% (11/24)*	68.42% (13/19) *
	Emergency Cesarean	0% (0/17) *	12.50% (3/24) *	0% (0/19) *
Neonatal Outcomes	Mean Birth Weight (g)	3284 ± 458	3296 ± 476	3373 ± 624
	Birth weight < 2500 g	0% (0/28) *	5.26% (2/38) *	8.33% (2/24) *
	Birth weight > 4000 g	7.14% (2/28) *	7.89% (3/38) *	12.50% (3/24) *
	Malformations	0	0	1 (ureteral stenosis)
Postpartum	Breastfeeding	66.67% (8/12) *	64.29% (9/14) *	62.50% (10/16) *
	Cessation to resume therapy	37.50% (3/8) *	42.86% (3/7) *	---

*Percentages are calculated based on the total number of cases with available data for each specific parameter. **The “Continuation / Modification” group included 13 pregnancies (43.3%) that required continuous medical treatment throughout gestation, while the remaining cases underwent pharmacological switch or late re-introduction to control disease activity

### Pre-conception withdrawal (Group 1)

Medical therapy was discontinued prior to conception in 40 pregnancies (30.53%) as part of planned pregnancy management [5, 7, 13, 14, 24, 27, 35, 38, 39, 46, 49]. Detailed therapeutic regimens are summarized in Table 4. The mean interval of drug withdrawal prior to conception was 5.4 ± 5.7 months [14, 24, 27, 38, 46, 50]. In three pregnancies achieved via ART, withdrawal strategies were tailored to the specific clinical context: therapy was discontinued between 14 days (octreotide LAR) [27] and 3 days (PEG) before embryo transfer [46].

**Table 4:** Distribution of medical therapies according to management strategy

Medical Therapy	Group 1 (*n* = 40)	Group 2 (*n* = 47)	Group 3 (*n* = 30)
First-generation SRLs	55.00% (22/40)	65.96% (31/47)	66.67% (20/30)
			*- Throughout pregnancy* 84.62% (11/13)
			- *Modification* 14.29% (1/7)**
Pasireotide (Mono/Combos)***	0% (0/40)	4.26% (2/47)*	3.33% (1/30)
			*- Throughout pregnancy* 7.69% (1/13)*
			- *Modification* 0% (0/7)**
First-gen SRLs + Dopamine Agonist	32.50% (13/40)	21.28% (10/47)	16.67% (5/30)
			*- Throughout pregnancy* 0% (0/13)
			- *Modification* 57.14% (4/7)**
Pegvisomant (Mono/Combos)***	10.00% (4/40)	8.51% (4/47)	13.33% (4/30)
			*- Throughout pregnancy* 7.69% (1/13)
			- *Modification* 28.57% (2/7)**
Other (e.g. SRL Combos)***	2.50% (1/40)	0% (0/47)	0% (0/30)
			- *Throughout pregnancy* 0% (0/13)
			- *Modification* 0% (0/7)**

The “Continuation / Modification / Re-intro” group includes a specific sub-analysis of 13 pregnancies with therapy maintained throughout gestation and 7 pregnancies where the regimen was modified upon pregnancy confirmation. * One case of continuous pasireotide therapy resulted in a spontaneous abortion at 20 weeks. The other case in the Group 2 refers to the present clinical case (pasireotide + cabergoline). ** In the **Modification subgroup** (*n* = 7) the pre-existing regimen was adjusted as follows: **first-generation SRL** (*n* = 1), switched to bromocriptine monotherapy [43]; **SRL + Dopamine Agonist** (*n* = 4), transitioned to octreotide LAR monotherapy (*n* = 2) [7, 20], continued cabergoline only (*n* = 1) [2] or switched to bromocriptine monotherapy (*n* = 1) [43]; **pegvisomant combinations** (*n* = 2), one case transitioned to octreotide LAR monotherapy [7] and one was converted to pegvisomant monotherapy [51]. ***Pasireotide (Mono/Combos): Includes patients receiving pasireotide as a single agent (monotherapy) [7, 50] or in combination with DA, as seen in our present case. **Pegvisomant (Mono/Combos)**: Refers to the use of pegvisomant either as monotherapy [25, 28, 38, 46] or in dual therapy regimens (combined with SRLs [5, 7, 35, 46] or DA [46, 51]). **Other (e.g.**,** SRL Combos)**: Comprises the association of different SRLs, such as octreotide and lanreotide [49]

#### Clinical course and maternal complications

Most patients (66.67%; 10/15) reported no significant symptoms during pregnancy [7, 38, 39, 49]. Clinical worsening, primarily characterized by headache, was described in 4 pregnancies (26.67%) [7, 39, 49]. Maternal complications included GDM (5.56%; 2/36), managed with dietary control alone [46, 49], and two episodes of pre-eclampsia occurring in the same patient during consecutive pregnancies [7].

#### Obstetric and neonatal outcomes

Two spontaneous abortions and one therapeutic abortion were recorded, the latter because of active acromegaly and severe headaches in the first trimester [5, 49]. This patient had a history of a 3.8 cm macroadenoma and, following the termination, required a second surgical resection and radiotherapy to achieve disease control [49]. Excluding these cases, as detailed in Table 3, most pregnancies (94.44%; 17/18) reached term with no emergency cesarean sections recorded [7, 14, 24, 27, 38, 39, 46, 49]. All neonates were born alive, with a mean birth weight of 3284 ± 458 g and no reported congenital malformations.

#### Lactation

Breastfeeding was initiated in most cases (66.67%; 8/12) [24, 27, 39, 46]. In one case, breastfeeding was discontinued to resume PEG therapy due to persistent headache [46]. Two other patients ceased breastfeeding after 4–5 months to restart octreotide LAR due to active disease [39].

### Discontinuation upon pregnancy diagnosis (Group 2)

In most cases (*n* = 47; 35.88%), medical therapy was discontinued immediately after pregnancy confirmation at a mean gestational age of 6.3 ± 3.7 weeks (range 2–15) [Our Case, 11, 12, 15, 18, 23, 25, 26, 32, 40, 42, 46]. Therapeutic regimens for this cohort are detailed in Table 4.

#### Clinical course and maternal complications

While 72.22% (26/36) reported no significant symptoms [Our Case, 2, 25, 26, 37–42, 46], clinical worsening occurred in 6 cases (16.67%) [12, 25, 38, 50]. Headache was reported in 4 cases [38, 50]. The correlation between symptoms and tumor volume was variable. Petyt et al. provided detailed MRI evidence of progressive adenoma enlargement (from 16 × 10 mm to 23 × 21 mm) with cavernous sinus invasion throughout pregnancy [50]. Similarly, Montini et al. described a symptomatic re-expansion of a known macroadenoma at 39 weeks, leading to severe intracranial hypertension [12]. Conversely, clinical progression did not always align with radiological findings, as demonstrated by Qureshi et al., who reported visual field deterioration at 30 weeks despite a stable pituitary mass on MRI [25]. Symptom improvement, such as regression of acral volume, was noted in 11.11% (4/36) of pregnancies [13, 22, 29, 32]. Regarding maternal complications, GDM was diagnosed in 18.75% (6/32) of evaluable cases [Our Case, 12, 25, 38, 46]. In 3 cases dietary control was insufficient and insulin therapy was required [Our Case, 12, 25]. Gestational hypertension occurred in 8.11% (3/37) of cases [38, 41, 46], progressing to pre-eclampsia in one instance [38].

#### Obstetric and neonatal outcomes

 Two therapeutic abortions were elected due to concerns about early octreotide exposure, with the first at 15 weeks of gestation [23] and the second at week 6 [41]. Excluding these, 88.89% (32/36) of pregnancies reached term [2, 12, 14, 18, 22, 24–26, 29, 32, 37–39, 41, 44, 46, 50], while pre-term delivery occurred in 8.33% (3/36) [11, 38, 42] and post-term delivery in 2.78% (1/36) [33]. Delivery mode was documented for 24 pregnancies: 41.67% (10/24) were spontaneous vaginal deliveries [2, 15, 22, 24, 29, 32, 33, 39, 41, 50], while the remainder required cesarean section. Elective cesarean Sect. (45.83%; 11/24) [2, 24, 25, 37, 41, 44, 46] were indicated for reasons including GDM and visual field alterations [25] or twin IVF pregnancies [46]. Emergency cesarean Sect. (12.50%; 3/24) [Our case, 11, 12] were performed for critical indications such as severe maternal intracranial hypertension with fetal respiratory distress [12] or impending fetal asphyxia [11]. Mean birth weight of newborns was 3296 ± 476 g. Fetal macrosomia was observed in 3 cases (7.89%) [13, 18]. Conversely, a low-birthweight (LBW) was recorded in 2 cases (5.26%): one case involved a neonate from a twin pregnancy [46], while the other was appropriate for the gestational age of 35 weeks [38]. No congenital malformations were reported in any case.

#### Lactation

Breastfeeding was initiated in 64.29% (9/14) of evaluable cases [Our Case; 2, 13, 22, 32, 37, 39]. While two patients maintained lactation after resuming octreotide LAR [22, 32], three others, including our index case, discontinued breastfeeding to restart treatment due to symptomatic recurrence or radiological evidence of tumor expansion [37, 39].

### Continuation of therapy during gestation (Group 3)

In 30 cases (22.90%), medical therapy was maintained throughout gestation, modified, or introduced later to control active disease [2, 5, 7, 13, 16, 19–21, 28, 31, 34, 35, 39, 43, 47, 51], as detailed in Table 4. Therapy was continued throughout gestation in 13 pregnancies (43.33%) [5, 7, 13, 21, 28, 31, 34, 47]. Continuous medical therapy during gestation was driven by both clinical indications and patient preference. Primary reasons included the management of refractory headache [13, 21, 31] and the prevention of symptomatic tumor expansion or visual compromise [5, 19, 34]. Additional factors were the need to maintain biochemical stability in refractory disease [28] and delayed pregnancy diagnosis [7, 47]. In 7 cases (23.33%) the pre-existing pharmacological regimen was modified upon pregnancy confirmation [2, 7, 20, 43, 51], typically by transitioning from combination therapy to monotherapy or switching to dopamine agonists (DAs) as detailed in Table 4. In 2 cases (6.67%), medical therapy was introduced de novo during gestation due to clinical reactivation [16, 39]. Mozas et al. reported a delayed pregnancy diagnosis in a patient receiving short-acting octreotide until week 10 and undergoing transsphenoidal surgery at week 16. Two months post-surgery, pregnancy was diagnosed and due to the post-surgical clinical reactivation (headache and retro-orbital pain), bromocriptine therapy was initiated and continued until delivery [16]. Assal et al. described the reintroduction of octreotide LAR at week 12 due to clinical reactivation (headache and hyperhidrosis) [39]. In 8 cases (26.67%) pharmacological therapy was maintained for limited periods (ranging from 12 to over 20 weeks) before discontinuation [7, 35].

#### Clinical course, maternal and neonatal outcomes

Significant maternal complications were documented by Jallad et al. in a single patient who experienced, across two distinct pregnancies (both involving octreotide LAR discontinuation at week 16), an exacerbation of pre-existing diabetes and progressed to pre-eclampsia. These gestations resulted in one infant presenting with ureteral stenosis, the only congenital malformation recorded, and one LBW neonate (2.25 kg). A second case of LBW (2.1 kg) occurred in a patient treated with octreotide LAR until week 20, who delivered preterm at 35 weeks [7]. Three cases of spontaneous abortion were recorded, one involving continuous pasireotide exposure (week 20) [7] and two following a switch to bromocriptine monotherapy (weeks 6 and 8) [43]. The remaining 27 pregnancies resulted in live births, with a mean birth weight of 3373 ± 624 g.

#### Lactation

Breastfeeding was successful in most evaluable cases (Table 3). Notably, lactation was maintained without complications in patients receiving subcutaneous octreotide [7, 13], octreotide LAR [39] or PEG [51]. One patient reported prolonged breastfeeding for 12 months while on medical therapy with first-generation SRL [47].

## Discussion

Pregnancy in acromegaly is increasingly reported in clinical practice, due to improved disease control and advancements in fertility treatments. Ideally, conception should be planned with prior tumor and hormonal control [6, 7, 39]. However, unplanned pregnancies frequently occur in women with both controlled and active disease, and occasionally acromegaly is diagnosed for the first time during pregnancy [17, 36, 45, 48].

This review evaluates 135 pregnancies, providing an up-to-date assessment of SRL and PEG safety. Notably, our presented clinical case represents the third literature report of pasireotide exposure during pregnancy [7, 50].

According to the current guidelines, treatment with long-acting SRL or PEG should be discontinued approximately two months before conception, reserving therapy for selected cases of tumor growth or severe headaches [9]. Our cohort largely reflects this standard, with most patients managed through pre-conception withdrawal (Group 1) or discontinuation upon pregnancy diagnosis (Group 2). However, our analysis highlights a subset of patients (Group 3) who required continuation, modification or re-introduction of medical therapy. Specifically, continuous treatment throughout gestation was required in 13 pregnancies [5, 7, 13, 21, 28, 31, 34, 47], primarily for tumor control and refractory headaches [5, 34]. We identified some cases requiring reintroduction or modification of treatment during gestation, including different therapeutic regimens [2, 7, 16, 20, 39, 43, 51]. This variability underscores the lack of a uniform strategy and emphasizes the need for individualized treatment in severe cases [8]. In some instances, clinicians switched to DA therapy [2, 16, 43] given its better pregnancy safety profile despite lower efficacy compared to SRL/PEG [9, 10, 39]. Overall, in our cohort 16.8% (22/131) of patients diagnosed prior to pregnancy required treatment continuation or resumption. This slightly exceeds the previously reported 12% [8], likely because our cohort selectively focused on SRL/PEG-exposed pregnancies.

The low rate of therapy continuation reflects physiological pregnancy adaptations. Elevated estrogen levels induce hepatic GH resistance, ensuring clinical stability during early phase of pregnancy despite autonomous tumoral GH secretion. This, combined with the residual effects of pre-conception long-acting SRL, supports safe withdrawal of medical therapy in most cases [52]. Indeed, symptom exacerbation, predominantly characterized by headaches, was observed in 26.67% of Group 1 [7, 39, 49] and 16.67% of Group 2 [12, 25, 38, 50]. Notably, 11.1% of Group 2 experienced clinical improvement, an outcome primarily described in women who had inactive disease prior to gestation [13, 22, 29, 32]. Estrogen-mediated lactotroph hyperplasia during pregnancy increases the risk of mass effects (e.g., visual impairment, severe headaches), particularly in pre-existing macroadenomas [52]. We observed rare radiologically confirmed tumor growth, primarily in Group 2 [12, 50]. Literature reports symptomatic expansion in < 9% of cases, with an increased risk for unoperated macroadenomas [8].

Cardiometabolic complications associated with active acromegaly, such as hypertension, impaired glucose tolerance and diabetes mellitus, increase the risk of maternal-fetal adverse events. Because pregnancy is inherently an insulin-resistant state, these pre-existing metabolic conditions frequently worsen [52]. GDM occurred in 10.91% (12/110) of our cohort. While lower than the 14.0% global prevalence, it slightly exceeds the standardized estimates for Europe (7.8%) and North America (7.1%) [53] and the 9% previously reported in acromegaly [8]. Notably, GDM incidence peaked at 18.75% in Group 2, suggesting that therapy withdrawal upon pregnancy diagnosis may exacerbate metabolic complications. Pre-eclampsia affected 5.88% (7/119) of pregnancies, a rate in the upper range of the general population (2–8%) [54], yet perfectly aligned with the 6% rate previously observed in acromegalic cohorts [8]. Ultimately, these hypertensive and metabolic complications led to preterm delivery, low birth weight (LBW), and cesarean Sects [7, 38].

The frequency of spontaneous abortions in our cohort is low (4.59%), significantly lower than the estimated 25% reported for the general population in the first trimester [53]. A late miscarriage at 20 weeks occurred during pasireotide therapy; however, this was likely attributed to the patient’s history of recurrent pregnancy loss and uncontrolled disease [7]. Three elective terminations occurred due to active disease [49] or early octreotide exposure [23, 41]. Most pregnancies reached full term (91.03%), with a consistently low rate of preterm births across all therapeutic groups [7, 11, 38, 42]. Elective cesareans were common (53.13% overall; 68.42% in Group 3), driven by two main clinical factors. First, in patients with large macroadenomas, the Valsalva maneuver during vaginal delivery increases intracranial pressure, which can potentially trigger pituitary apoplexy [24, 39]. Second, the presence of maternal complications - such as severe hypertension, pre-eclampsia, or GDM - frequently guides the clinical decision toward a cesarean delivery to minimize systemic obstetric risks [2, 7]. Medical treatment with SRL during pregnancy has been associated with LBW [20, 41]. However, our data suggest that LBW in acromegalic pregnancies is more likely secondary to obstetric factors (e.g., twin gestation, pre-eclampsia, preterm delivery) rather than a direct drug effect [7, 38, 46]. Furthermore, the only congenital malformation identified in our cohort was a single case of ureteral stenosis in a Group 3 infant. Given the mother’s complex clinical course, characterized by worsening diabetes and pre-eclampsia, this malformation appears to be an isolated event rather than a teratogenic effect of medical therapy [7].

The European Society of Endocrinology Guidelines [55] advise avoiding treatment with SRL and PEG during breastfeeding, considering individual circumstances, due to limited available data. However, while SRL are excreted in breast milk, their oral bioavailability for the infant is likely negligible; in addition, PEG secretion is considered undetectable, since polyethylene glycol is not excreted into breastmilk [56]. In our cohort, some patients successfully breastfed while on medication [7, 13, 22, 32, 39, 51], including one case of prolonged breastfeeding (12 months) during SRL therapy with no adverse effects reported [47]. These findings suggest breastfeeding remains a feasible, monitorable option despite requiring postpartum treatment.

This review has limitations. As a narrative review of a rare condition, the available evidence is heterogeneous and largely derived from case reports and small retrospective series; the included studies differed in design, completeness of reporting and outcome definitions, and no formal quality or risk-of-bias assessment was performed. The pooled proportions reported here are therefore descriptive and should be interpreted with caution rather than as precise estimates of incidence or risk.

## Conclusions

In conclusion, pregnancy in acromegaly is generally characterized by clinical stability, supporting the safety of treatment withdrawal upon conception in most patients. When gestational therapy is required due to clinical worsening, maternal and fetal complications remain infrequent and are primarily driven by pre-existing acromegaly-related comorbidities rather than direct drug toxicity. Consequently, pre-conception counseling is essential to optimize metabolic status before pregnancy.

Notably, we report a rare case of pasireotide exposure during early gestation with no adverse maternal or neonatal outcomes. Given the limited literature, further studies are necessary to confirm the safety profile of second generation SRL in pregnancy.

## Data Availability

Data are available on reasonable request.
